# Correction: Comparison between single and serial computed tomography images in classification of acute appendicitis, acute right-sided diverticulitis, and normal appendix using EfficientNet

**DOI:** 10.1371/journal.pone.0294794

**Published:** 2023-11-17

**Authors:** The PLOS ONE Staff

There are errors in the Funding section. The correct Funding statement is: This study was supported by the Gacheon Gil Medical Center (FRD2022-12), and by the GRRC project (GRRC-Gacheon 2020 (B02)) of Gyeonggi-do.

The publisher apologizes for the error.
