# Supplementary material for: Comparison between single and serial computed tomography images in classification of acute appendicitis, acute right-sided diverticulitis, and normal appendix using EfficientNet
Source: PLoS One. 2023 May 24;18(5):e0281498. doi: 10.1371/journal.pone.0281498 (PMC10208462; doi:10.1371/journal.pone.0281498)

S1. Appendix. Five-fold cross-validation (a-b) single methods (c-d) RGB methods in classification among acute appendicitis, acute diverticulitis, and normal appendix using EfficientNet (no lesion means normal appendix)


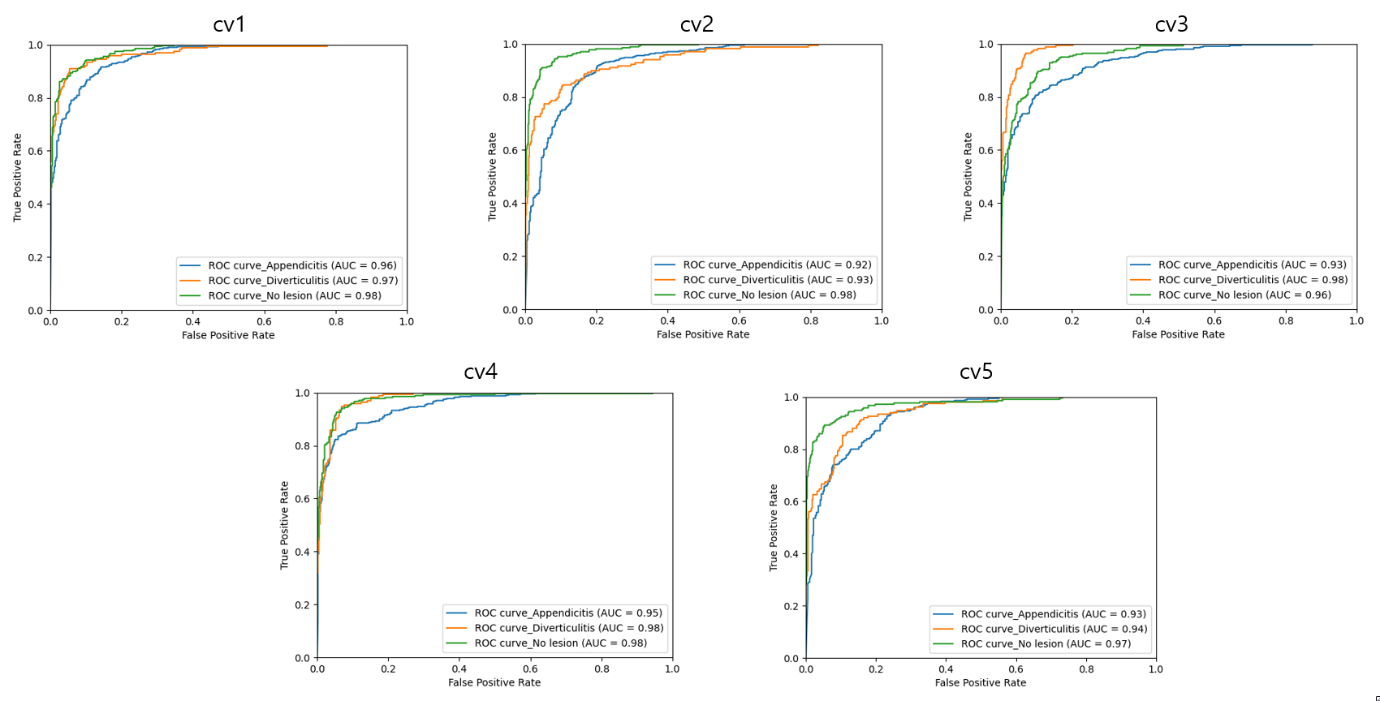
(a)

(b)


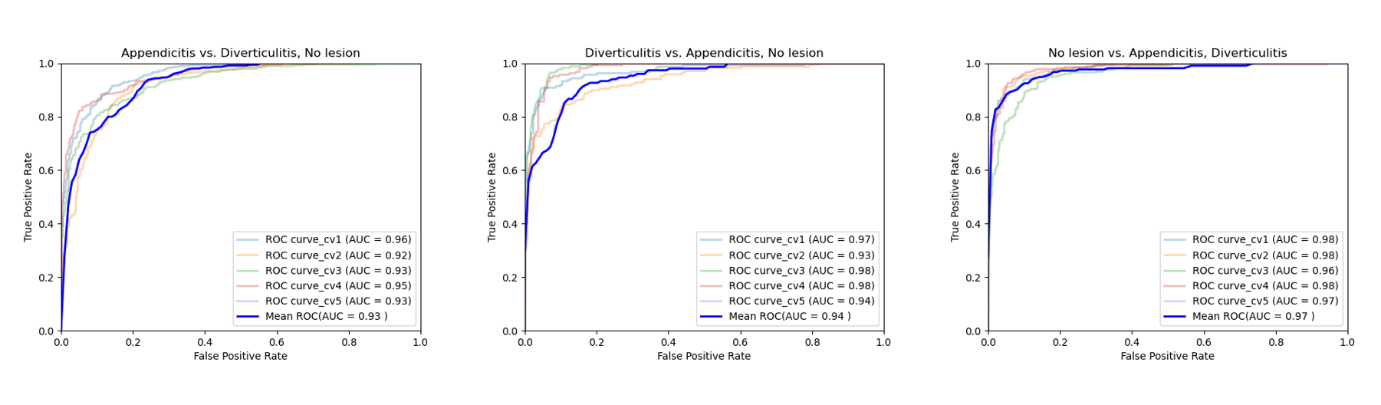


(c)


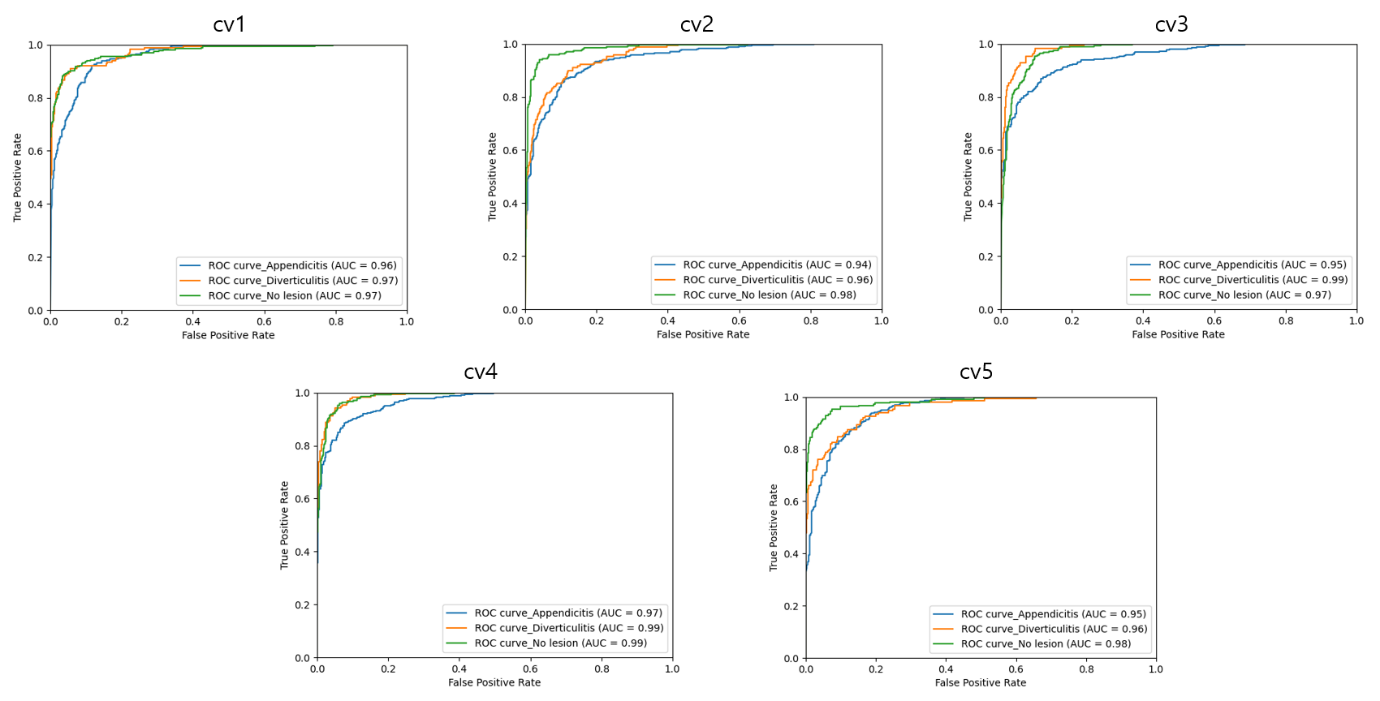


(d)


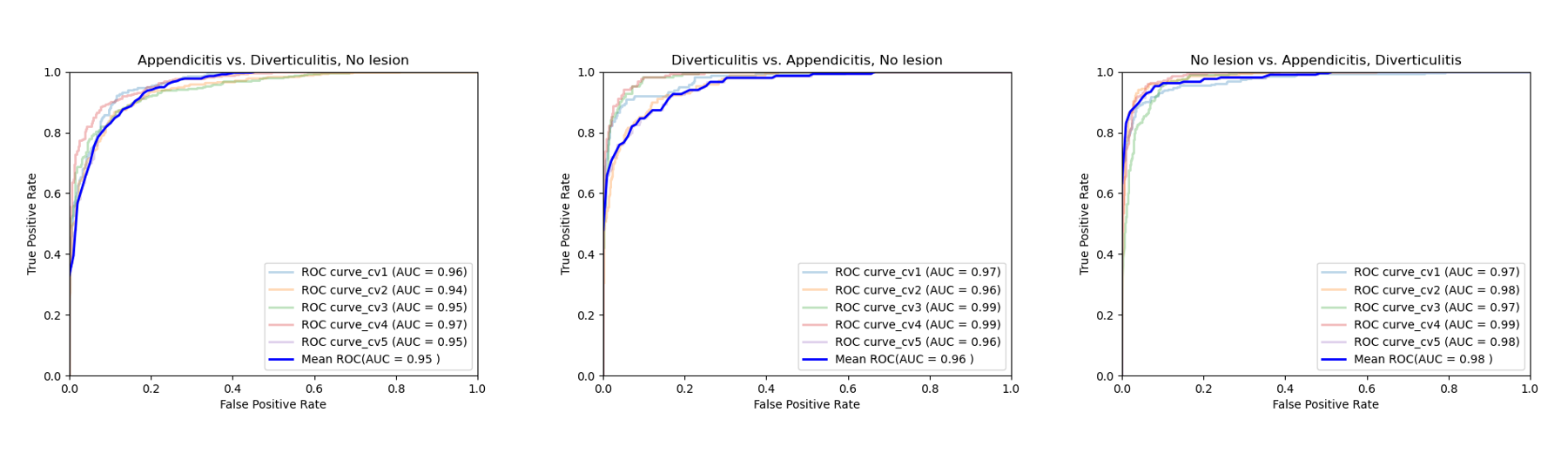

Supplement: S1 Appendix — (a,b) Single methods (c,d) RGB methods for classification of acute appendicitis, acute diverticulitis, and normal appendix using EfficientNet. (DOCX) [file pone.0281498.s002.docx]
